# Genetic Drivers of Multidrug Resistance in *Candida glabrata*

**DOI:** 10.3389/fmicb.2016.01995

**Published:** 2016-12-15

**Authors:** Kelley R. Healey, Cristina Jimenez Ortigosa, Erika Shor, David S. Perlin

**Affiliations:** Public Health Research Institute, Rutgers Biomedical and Health Sciences, New Jersey Medical SchoolNewark, NJ, USA

**Keywords:** antifungal resistance, *Candida glabrata*, DNA repair, *MSH2*, echinocandins, triazoles, karyotyping, MLST genotyping

## Abstract

Both the incidence of invasive fungal infections and rates of multidrug resistance associated with fungal pathogen *Candida glabrata* have increased in recent years. In this perspective, we will discuss the mechanisms underlying the capacity of *C. glabrata* to rapidly develop resistance to multiple drug classes, including triazoles and echinocandins. We will focus on the extensive genetic diversity among clinical isolates of *C. glabrata*, which likely enables this yeast to survive multiple stressors, such as immune pressure and antifungal exposure. In particular, over half of *C. glabrata* clinical strains collected from U.S. and non-U.S. sites have mutations in the DNA mismatch repair gene *MSH2*, leading to a mutator phenotype and increased frequencies of drug-resistant mutants *in vitro*. Furthermore, recent studies and data presented here document extensive chromosomal rearrangements among *C. glabrata* strains, resulting in a large number of distinct karyotypes within a single species. By analyzing clonal, serial isolates derived from individual patients treated with antifungal drugs, we were able to document chromosomal changes occurring in *C. glabrata in vivo* during the course of antifungal treatment. Interestingly, we also show that both *MSH2* genotypes and chromosomal patterns cluster consistently into specific strain types, indicating that *C. glabrata* has a complex population structure where genomic variants arise, perhaps during the process of adaptation to environmental changes, and persist over time.

## Epidemiology of fungal infections and treatment options

Fungi infect billions of people around the world each year. Although most fungal infections remain superficial, millions develop invasive mycoses, which have alarmingly high mortality rates ranging from 30 to 90%, even with antifungal treatment (Pfaller and Diekema, 2007; Nivoix et al., 2008; Wingard et al., 2008; Roilides et al., 2009). According to the CDC and WHO, the number of deaths caused by fungal infections is comparable to the numbers attributed to the world's deadliest diseases, including malaria and tuberculosis. The majority of invasive fungal infections (IFIs) and deaths occur in immunocompromised individuals and are caused by species of *Candida, Aspergillus*, or *Cryptococcus* (Pfaller and Diekema, 2007). While *Aspergillus* and *Cryptococcus* are acquired from the environment, *Candida* is a human commensal and most infections arise endogenously. *Candida* species account for most mucosal and invasive fungal infections worldwide (Pfaller et al., 2006) and are associated with significant healthcare costs (Zaoutis et al., 2005; Pfaller and Diekema, 2010). Approximately 50% of candidemia cases in the U.S. are caused by *C. albicans* (Hajjeh et al., 2004; Azie et al., 2012). However, over the past 20 years there has been a shift toward non-albicans *Candida* species (Pfaller and Diekema, 2004; Diekema et al., 2012; Lockhart et al., 2012), and the identity of leading non-albicans species causing disease varies upon geographical location. In the U.S., *C. glabrata* accounts for ~25% of infections, followed by *C. parapsilosis* (~15%) and *C. tropicalis* (~10%) (Pfaller et al., 2011a,b; Azie et al., 2012; Lockhart et al., 2012). Decreases in *C. albicans* infections have been complemented by increases in *C. glabrata* infections in most U.S. cities (Lockhart et al., 2012). The reason for the steep increase in reported *C. glabrata* infections is not known, although advances in diagnostic methods, increases in the elderly population, geography, and widespread fluconazole use (see below) have been proposed to play roles (Pfaller et al., 2006, 2009; Diekema et al., 2012).

Patients undergoing procedures such as stem cell or organ transplantation, surgery, or cancer treatment are at high risk for developing life-threatening IFIs. Consequently, such patients are commonly placed on antifungal prophylaxis with either triazole or echinocandin drugs. The triazole class of antifungal drugs targets the biosynthesis of ergosterol, which is a critical component of fungal cell membranes, while the echinocandins block the biosynthesis of beta-1,3-glucan, a fundamental structural component of the cell wall. Both drug classes are recommended first-line therapy for a variety of IFIs caused by *Candida* species (Pappas et al., 2016). A consequence of the widespread use of triazole antifungals (e.g., fluconazole) for prophylaxis or therapy is the selection of *Candida* species that readily develop resistance, such as *C. glabrata* (Lortholary et al., 2011). Approximately 20–30% of *C. glabrata* strains, but less than 5% of *C. albicans* strains, exhibit fluconazole resistance in the U.S. (Castanheira et al., 2014). *C. glabrata* readily develops cross-resistance to azoles, including fluconazole, itraconazole, voriconazole and posaconazole, which is often associated with upregulation of ATP-binding cassette (ABC) transporters, such as *CDR1* and *SNQ2*, that effectively pump the drugs out of the yeast cell (Sanglard et al., 1999, 2001; Izumikawa et al., 2003; Torelli et al., 2008). Upregulation of these drug efflux pumps is usually accompanied by a gain-of-function mutation in the transcription factor *PDR1* (Vermitsky and Edlind, 2004; Tsai et al., 2006; Vermitsky et al., 2006), prompting the recent development of a novel inhibitor that interferes with Pdr1 binding to the Mediator complex preventing transcription initiation (Nishikawa et al., 2016). Specific *pdr1* mutations in *C. glabrata* can also lead to a gain of fitness through enhanced adhesion and virulence (Ferrari et al., 2011; Vale-Silva et al., 2013, 2016). Additional mechanisms of triazole resistance, such as mutation of the drug target (*ERG11*; ergosterol biosynthesis) or mutation of genes (e.g., *ERG3*) that prevent the production of toxic intermediates caused by triazole action, have been identified in other *Candida* species (Cowen et al., 2014), yet rarely in *C. glabrata* (Hull et al., 2012).

In some settings, such as those involving hematologic malignancies, *C. glabrata* is isolated more often than *C. albicans*, making it the leading cause of IFIs (Hachem et al., 2008). Therefore, current treatment recommendations advise administration of an echinocandin to patients with known *C. glabrata* infections (Pappas et al., 2016). While the mechanism of echinocandin resistance is consistent across *Candida* species, the rates of resistance have increased the greatest (from 3 to 12%) in *C. glabrata* (Alexander et al., 2013) for reasons that are still largely unclear. Echinocandin resistance develops upon mutation of the catalytic subunits (Fks1/Fks2) that make up the echinocandin target enzyme, beta-1,3-glucan synthase. Mutations are generally found in the “hot spot” regions of either *FKS* gene and result in cross-resistance to all echinocandins (caspofungin, micafungin, and anidulafungin) (Perlin, 2015). Triazole-resistant clinical isolates (Pfaller et al., 2005; Messer et al., 2006) and laboratory strains (Niimi et al., 2006) that demonstrate increased drug efflux pump expression remain susceptible to the echinocandins in laboratory liquid assays. One study from 2003 (Schuetzer-Muehlbauer et al., 2003) did report reduced caspofungin susceptibility upon overexpression of the ABC efflux pump *CDR2* in *C. albicans*, although this occurred under specific testing conditions and the other echinocandins (anidulafungin and micafungin) were not yet available for testing. Overall, the echinocandins do not appear to be substrates for drug efflux pumps conferring azole resistance. Echinocandins are also effective against some *Candida* biofilms, which are normally refractory to triazole treatment (Kuhn et al., 2002; Katragkou et al., 2008). A third class of antifungals, the polyenes, directly targets ergosterol in the cell membrane disrupting cellular permeability; however, treatment of invasive *Candida* infections with the polyene amphotericin B is only recommended as a second-line agent due to toxicity issues (Pappas et al., 2016).

## The adaptive response

One factor that can influence the survival or persistence of fungi between the initial drug exposure and acquisition of resistance-conferring mutations is the adaptive response. Upon exposure to environmental stresses, such as temperature, ionic, oxidative, and osmolarity changes, and/or drug pressure, fungi activate stress responses to mitigate the harmful effects and avoid death. Of particular importance upon echinocandin exposure is the cell wall integrity (CWI) pathway, which regulates glucan synthesis through Rho1 and cell wall repair (Levin, 2005). Following cell wall stress, Rho1 activation leads to activation of protein kinase C (*PKC1*) and upregulation of the *FKS* genes. Targeting the CWI pathway through deletion of *PKC1* or activated MAP kinases (e.g., Cg*SLT2*) induces echinocandin hypersensitivity; while conversely, an increased activation of the pathway, such as through over-expression of Cg*SLT2*, reduces susceptibility (increases tolerance) of *C. glabrata* to echinocandins (Cota et al., 2008; Miyazaki et al., 2010a; Schwarzmuller et al., 2014). Induction of the CWI pathway in *Candida* species (Stevens et al., 2006; Walker et al., 2013), including *C. glabrata* (Cota et al., 2008), has been linked to increased production of other cell wall components, such as chitin, potentially compensating for the loss of beta-1,3-glucans. Genetic (Ueno et al., 2011; Schwarzmuller et al., 2014) or chemical (Miyazaki et al., 2010a) targeting of chitin synthase has been shown to increase *C. glabrata* susceptibility to echinocandins *in vitro*. Regulators of the CWI pathway, such as Hsp90 and calcineurin, can similarly influence the susceptibility of *C. glabrata* to echinocandin and triazole drugs (Singh et al., 2009; Miyazaki et al., 2010b). Other epigenetic mechanisms, such as chromatin modification, may impact drug activity; for example, histone deacetylase inhibitors act synergistically with antifungals (Garnaud et al., 2016). In addition to genetic and epigenetic changes, alterations in chromosomal structure may also influence survival *in vivo* and antifungal susceptibility; this will be discussed in greater depth in subsequent sections of this perspective. Tolerance mechanisms may “prime” cells, particularly *C. glabrata*, to better survive adverse conditions *in vivo* and, in turn, better equip fungi to survive antifungal pressure. Inversely, abrogating the adaptive response of *C. glabrata* may increase the killing (fungicidal index) efficacy of antifungals. Thus, a better understanding of how fungi control these adaptive mechanisms, particularity *in vivo*, and how fitness may be affected is warranted.

In addition to individual cell responses to drugs, which are determined largely by cell genotype, population responses may also play a role in drug tolerance. Although acquisition of a *pdr1* mutation is required for stable triazole resistance, studies have demonstrated the ability of *C. glabrata* subpopulations originating from a clonal population to develop heteroresistance to fluconazole through transient upregulation of efflux pumps (Claudino et al., 2009; Ben-Ami et al., 2016). Development of tolerance to fluconazole treatment at the population level may be one consequence of heteroresistance, which in turn, may allow the yeast to adapt and “buy time” until beneficial, stable mutations develop. As stated earlier, specific *pdr1* mutations can also enhance the fitness of *C. glabrata* and increase virulence through adhesion upregulation (Vale-Silva et al., 2016). *C. glabrata* heteroresistance was not observed in response to the echinocandins, which may reflect its fungicidal mechanism of action (at least *in vitro*) (Ben-Ami et al., 2016) or potential associated fitness costs. However, yeast are exposed to additional pressures *in vivo* that may induce some kind of transient heterotolerance to the echinocandins that is not necessarily measurable *in vitro*.

## The emergence of multidrug resistance in *C. glabrata*

While the individual mechanisms of *C. glabrata* triazole and echinocandin resistance have been extensively examined, the sudden emergence of strains that are resistant to both drug classes is surprising and alarming. Resistance to multiple antifungal classes, or multidrug resistance (MDR), has been reported most frequently in this fungus (Pfaller et al., 2012). A CDC/SENTRY Antimicrobial Surveillance Program study found that while none of the fluconazole-resistant *C. glabrata* isolates from 2001 to 2004 demonstrated echinocandin resistance, 11% of fluconazole-resistant isolates from 2006 to 2010 were also resistant to an echinocandin (Pfaller et al., 2012). Another study, performed at Duke Hospital in 2010, found that 14% of fluconazole-resistant isolates also exhibited resistance to at least one echinocandin and that 3.5% of all *C. glabrata* isolates were MDR (Alexander et al., 2013). Additionally, a survey of *C. glabrata* isolates obtained from a cancer hospital between 2005 and 2013 reported nearly 7% as MDR (Farmakiotis et al., 2014). As a consequence of current guidelines for prophylaxis and treatment, the majority of MDR *C. glabrata* strains have acquired resistance to triazole and echinocandin class antifungals, with rare isolates demonstrating resistance to amphotericin B (Farmakiotis et al., 2014; Cho et al., 2015). Collectively, the emergence of MDR in *C. glabrata*, although still uncommon, is a cause for grave concern given the fact that echinocandin and triazole use has increased significantly over the past 10 years (Pfaller et al., 2012), and the echinocandins are currently the preferred treatment for *C. glabrata* invasive infections. A better understanding of the mechanisms responsible for the emergence or perpetuation of MDR will help assess factors that can both prevent and overcome resistance.

Many groups, including ours, have attributed the observed increases in drug resistance rates to the haploid genome of *C. glabrata*. While this is most likely a factor, our recent studies have suggested that other prominent biological factors may contribute more profoundly to resistance emergence. Here, we discuss genetic mechanisms, both at the chromosomal and nucleotide levels, which can increase the mutability and/or genetic diversity of *C. glabrata*. We propose that these mechanisms allow strains of *C. glabrata* to create genetically diverse populations within the human host and improve the yeast's chances of surviving multiple stressors, including antifungal treatment. We show that nucleotide polymorphisms and chromosomal rearrangements vary widely in *C. glabrata* yet group reliably into strain types. In this perspective, we will both summarize previously published studies and present new data that support these claims.

## Genetic diversity and the mutator phenotype

All cells contain multiple mechanisms dedicated to repairing DNA damage that can result from either exogenous (e.g., chemicals or radiation) or endogenous (e.g., reactive oxygen radicals or DNA polymerase errors) sources. In eukaryotes these mechanisms include DNA-damage checkpoints and several highly conserved DNA repair systems, including double-strand break repair (DSBR), base-excision repair (BER), nucleotide-excision repair (NER), and mismatch repair (MMR). Defects in these mechanisms are often associated with increased mutation rates or genome rearrangements, and in humans they are associated with a predisposition to specific cancers (Hakem, 2008). Thus, defects in DNA repair may also underlie the emergence of drug-resistant variants in fungal pathogens such as *C. glabrata*. DNA repair has been extensively studied in the model organism *Saccharomyces cerevisiae*. Yet, its role in fungal pathogens, especially in connection to emergence of antifungal resistance, has only begun to be addressed.

One study investigated the involvement of DNA repair mechanisms in drug resistance in *C. albicans* and demonstrated that mutants of MMR and DSBR, but not BER or NER, lead to increased appearance of fluconazole-resistant colonies (Legrand et al., 2007, 2008). Recently, we examined the role of DNA repair in *C. glabrata* (Healey et al., 2016). We discovered that over half (55%) of all *C. glabrata* isolates (susceptible and resistant) recovered from patients contain mutations within the MMR gene *MSH2*. Strains with specific *msh2* mutations exhibit a higher frequency of emergence of resistance *in vitro*, and deletion of *MSH2* causes elevated propensity to breakthrough antifungal treatment in a mouse model of gastrointestinal (GI) colonization. Furthermore, analysis of serial clinical isolates demonstrated that *msh2* mutations pre-date the emergence of antifungal resistance. Interestingly, *MSH2* genotypes appear to be geographically dependent. The overwhelming majority of strains (75 of 76) that exhibit *msh2-E231G/L269F* or *msh2-P208S/N890I* were recovered at U.S. based medical centers. Strains containing either *msh2-V239L/A942T* or *msh2-V239L* were identified in both U.S. and non-U.S. (Switzerland, Qatar, and India) centers, although these genotypes were predominant (95 of 125) among the *msh2* mutant alleles identified from the non-U.S. centers. These geographical differences are most likely a result of population and/or treatment differences (see “Strain type” section below). Our study suggests that *msh2* mutation is a driver of the acquisition of antifungal resistance in *C. glabrata*. Whether other fungi exhibit similar mechanisms of genetic diversity through MMR remains to be addressed.

Through *in vivo* competition assays utilizing both GI colonization and bloodstream infection mouse models, we have demonstrated that complete loss of MMR function (*msh2*Δ) impacts fitness of *C. glabrata* in the host (Healey et al., 2016). However, strains exhibiting one of the aforementioned *msh2* mutations lead to only partial loss-of-function (data not shown) and may not suffer from the extremely high mutation rate and fitness defect observed with *msh2*Δ. Additional mechanisms of DNA repair, including other MMR genes, and other cellular systems responsible for genome integrity maintenance, such as DNA-damage checkpoints, may also influence the mutagenic potential of *C. glabrata* clinical strains.

## Genetic diversity of chromosome structure

Normally, all chromosomes in a cell are present at the same copy number; however, deviations from this norm, aka aneuploid isolates, have been reported in fungal pathogens (Bennett, 2010; Gerstein et al., 2015). A number of studies have suggested that variations in chromosome organization and copy number can be advantageous to fungal cells under certain conditions and may provide a route for rapid adaptation to stress, such as the host immune response or antifungal drugs. In *C. albicans*, chromosomal rearrangements (copy number variation, loss of heterozygosity, translocations, chromosome truncations) occur in response to stresses such as heat shock, host-pathogen interactions, and the presence of antifungal drugs (Selmecki et al., 2006; Bouchonville et al., 2009; Forche et al., 2009). As a result, the karyotypes (the number and size of chromosomes) between different isolates may vary drastically (Merz et al., 1988; Rustchenko-Bulgac, 1991; Pfaller et al., 1994). Even greater karyotypic variability has been observed in clinical isolates of *C. glabrata*. Karyotyping using pulsed-field gel electrophoresis (PFGE), which separates whole chromosomes, revealed that *C. glabrata* genome organization varies significantly among different isolates (Muller et al., 2009; Polakova et al., 2009). Similar to what has been described in *C. albicans*, the most frequent genome rearrangements identified were translocations of chromosomal arms and inter-chromosomal duplications, but formation of novel chromosomes was also reported (Muller et al., 2009; Polakova et al., 2009).

**Figure 1:**
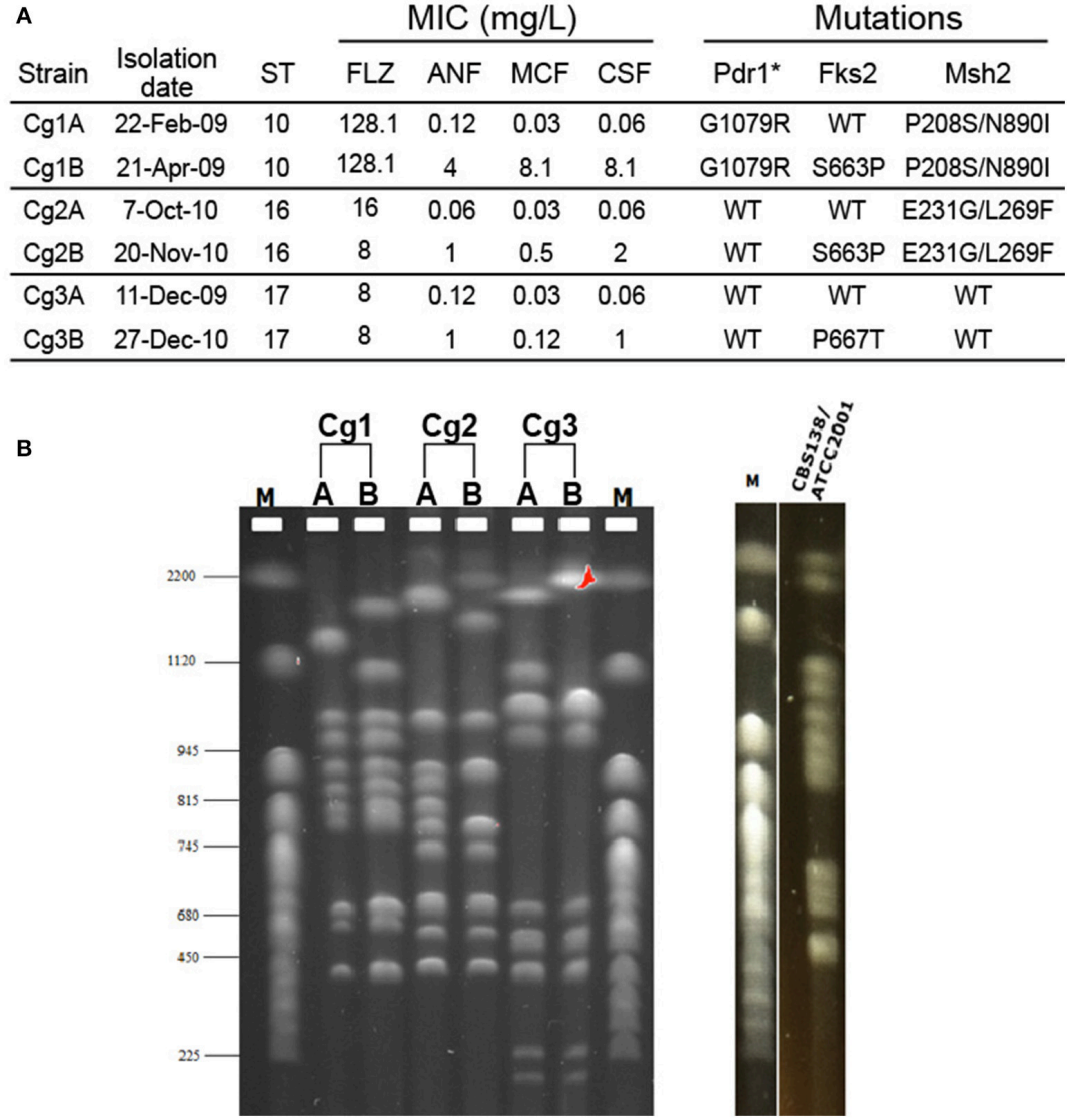
**Electrophoretic karyotypes of three pairs of serial ***C. glabrata*** clinical isolates. (A)** The minimum inhibitory concentrations (MIC), sequence type (ST), and genome mutations of two bloodstream isolates (A and B) from three individual patients (1, 2, and 3) are shown. **(B)** Karyotypes of the same isolates obtained with PFGE analysis. The karyotype of the *C. glabrata* reference strain CBS138/ATCC2001 is also shown. M: CHEF DNA chromosomal size marker (*S. cerevisiae* strain YNN295) (BIO-RAD). ANF, anidulafungin; MCF, micafungin; CSF, caspofungin; FLZ, fluconazole. ^*^Gain of function mutations.

Since genome plasticity has been connected with antifungal drug resistance in *C. glabrata* (Shin et al., 2007; Polakova et al., 2009), we analyzed the karyotypes of serial clinical *C. glabrata* isolates from our laboratory's strain collection. All strains were subjected to multi-locus sequence typing (MLST) analysis (Dodgson et al., 2003) to verify that each pair originated from the same lineage; indeed, both strains from each pair were of the same strain type (Figure 1A). MICs to fluconazole and the echinocandins were determined by the CLSI broth microdilution method (CLSI, 2012) and *FKS1, FKS2, PDR1*, and *MSH2* genes were sequenced for all isolates (Figure 1A). In each case, a bloodstream isolate was obtained before and after echinocandin therapy, and in each case the first isolate was echinocandin sensitive, while the second isolate had acquired echinocandin resistance and an *fks* mutation. One set of isolates exhibited resistance to fluconazole and contained a *pdr1* mutation. To determine whether these changes were also accompanied by large-scale chromosomal rearrangements, we performed PFGE. Karyotypes of the six isolates presented unique chromosomal profiles (Figure 1B). While strains isolated from the same patient were more similar to each other than strains isolated from different patients, even sequential isolates obtained from the same individual had distinct chromosomal bands, particularly among the larger chromosomes (Figure 1B). Thus, changes in chromosomal architecture occurred during microevolution of *C. glabrata* within the host, possibly in response to echinocandin treatment. Thus, genome rearrangements may be one mechanism used by *C. glabrata* to diversify the phenotypic properties of the population and promote adaptation under specific stress conditions.

## *C. glabrata* population structure: strain types are characterized by specific nucleotide polymorphisms and genome rearrangements

**Figure 2:**
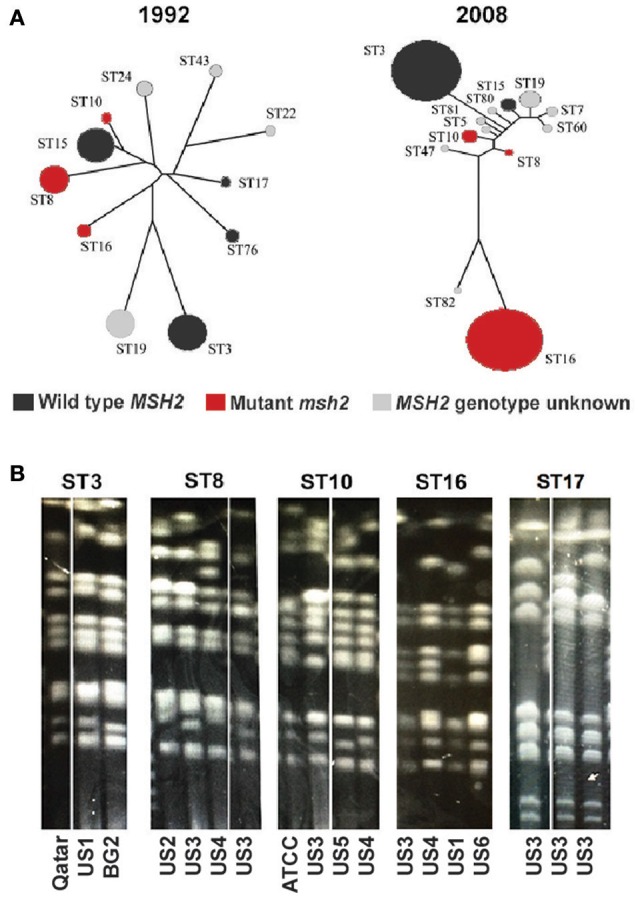
**Temporal changes and chromosomal patterns associated with ***C. glabrata*** strain type. (A)** The population structure of *C. glabrata* isolates obtained from candidemia patients in Atlanta hospitals during the indicated years. Each circle represents a particular ST and the *msh2* profile of each ST is indicated by color (adapted from Lott et al., 2010). **(B)** Strains belonging to the same ST have similar chromosome patterns as determined by pulse field gel electrophoresis (PFGE). Chromosome configurations for strains of five different STs are shown and the origins of every clinical isolate are indicated below. Laboratory strains (BG2 and ATCC) or isolates recovered from individual patients at six separate U.S. medical centers (US1-6) or a medical center in Qatar were analyzed.

As described above, *C. glabrata* clinical isolates have highly variable chromosomal profiles. It has been reported that this fungus also shows genetic variability at the level of genomic nucleotide sequence (Lin et al., 2007). A MLST scheme based on six different loci has thus far identified over 80 different *C. glabrata* sequence types (STs, or clades) (Dodgson et al., 2003) and our preliminary analysis indicates that this number is likely much higher. For instance, MLST typing of 48 clinical strains identified 6 strains containing novel MLST alleles or novel configurations of previously reported alleles (data not shown). We also found that different *msh2* alleles (see above and Figure 1A) are characteristic of distinct STs/clades, indicating that different STs may have different propensity toward mutability and acquiring drug resistant gene variants. This conclusion is significant because the distribution of *C. glabrata* STs varies both by geography and over time (Dodgson et al., 2003; Lott et al., 2010). For instance, Figure 2A shows that *C. glabrata* ST distribution in Atlanta area hospitals changed significantly between 1992 and 2008, the time period that includes the introduction of echinocandins (Lott et al., 2010). One noteworthy change is the increased prevalence of ST16, which carries an *msh2* mutation and may therefore acquire drug resistance at elevated rates.

The observation that different *C. glabrata* STs carry different *msh2* alleles makes it highly likely that other important determinants of drug resistance or virulence also vary among STs. A straightforward way to identify such determinants is by genome sequencing. However, the great degree of genomic rearrangement among strains presents a barrier to typical shotgun sequencing approaches that use a reference strain as template. Indeed, while the genome of a reference *C. glabrata* laboratory strain (CBS138) has been sequenced, we were not able to use that strain's genomic sequence as a template for assembling the sequences of other strains, e.g., various clinical isolates, due to the high karyotypic variability among strains (Muller et al., 2009; Polakova et al., 2009; Bader et al., 2012; Figure 1B). Thus, it will be necessary to assemble the genomes of individual clinical *C. glabrata* isolates *de novo* using techniques such as optical restriction mapping and long read DNA sequencing (Neely et al., 2011; Rhoads and Au, 2015).

We also examined the chromosomal architecture of strains belonging to different STs. Because it was originally proposed that chromosomal rearrangements in *C. glabrata* arise as part of the virulence process or adaptation to drug pressure and emergence of resistance (Polakova et al., 2009), we expected that there would be no correspondence between ST identity and chromosomal configuration. However, we were surprised to discover similarities in chromosomal architecture among strains belonging to the same STs (Figure 2B). Together with data presented above (Figure 1B), this observation suggests that chromosomal variants of *C. glabrata* both exist as commensal populations, where different STs are characterized by specific chromosomal configurations, and arise de novo, perhaps in response to pressure from the host and antifungal drug treatment.

## Conclusion

Accumulating evidence suggests that both increasing numbers of immunocompromised patients and the widespread use of triazoles, particularly fluconazole, have facilitated an increased prevalence of *C. glabrata* infections and more alarmingly, the emergence of multidrug resistant strains. Here we have summarized the current understanding of factors driving drug resistance in *C. glabrata*. Maximizing genetic diversity, a hallmark of *C. glabrata*, is most apparent by the high prevalence in the global population of mutator phenotypes mediated by mutations in *MSH2*. Additionally, new data is presented supporting the concept that *C. glabrata* has a surprisingly plastic and mutable genome. It appears that, in contrast to many organisms, *C. glabrata* can tolerate such a high degree of genome rearrangement and thrive under these circumstances, perhaps through acquisition of gain-of-fitness mutations. Continued tracking of *C. glabrata* strain types in medical centers around the globe will aid in the understanding of *C. glabrata* population structure and how pathogenesis and drug resistance are associated with different strains of *C. glabrata*.

## Ethics statement

All isolates analyzed in new studies presented here are a part of the Perlin laboratory historic strain collection and were previously obtained from mycology laboratories without patient identifiers.

## Author contributions

KH, CJ, ES, and DP wrote the paper. CJ, ES, and KH performed the experiments.

## Funding

This research was supported by grants from the NIH (AI109025) and Astellas Pharma (Echinocandin Reference Center) to DP and by an Arnold O. Beckman postdoctoral fellowship from the Arnold and Mabel Beckman Foundation to KH.

### Conflict of interest statement

DP has received research support and/or advised Merck, Astellas, Cidara, Synexis, Matinas, and Amplyx. The other authors declare that the research was conducted in the absence of any commercial or financial relationships that could be construed as a potential conflict of interest.
